# Neuroprotective effects of resistance physical exercise on the APP/PS1 mouse model of Alzheimer’s disease

**DOI:** 10.3389/fnins.2023.1132825

**Published:** 2023-04-06

**Authors:** Henrique Correia Campos, Deidiane Elisa Ribeiro, Debora Hashiguchi, Talita Glaser, Milena da Silva Milanis, Christiane Gimenes, Deborah Suchecki, Ricardo Mario Arida, Henning Ulrich, Beatriz Monteiro Longo

**Affiliations:** ^1^Laboratory of Neurophysiology, Department of Physiology, Universidade Federal de São Paulo, São Paulo, Brazil; ^2^Department of Biochemistry, Institute of Chemistry, University of São Paulo, São Paulo, Brazil; ^3^Instituto do Cérebro - ICe, Universidade Federal do Rio Grande do Norte, Natal, Brazil; ^4^Department of Psychobiology, Universidade Federal de São Paulo, São Paulo, Brazil

**Keywords:** Alzheimer’s disease, resistance exercise, locomotor activity, corticosterone, amyloid-beta precursor protein

## Abstract

**Introduction:**

Physical exercise has beneficial effects by providing neuroprotective and anti-inflammatory responses to AD. Most studies, however, have been conducted with aerobic exercises, and few have investigated the effects of other modalities that also show positive effects on AD, such as resistance exercise (RE). In addition to its benefits in developing muscle strength, balance and muscular endurance favoring improvements in the quality of life of the elderly, RE reduces amyloid load and local inflammation, promotes memory and cognitive improvements, and protects the cortex and hippocampus from the degeneration that occurs in AD. Similar to AD patients, double-transgenic APPswe/PS1dE9 (APP/PS1) mice exhibit Αβ plaques in the cortex and hippocampus, hyperlocomotion, memory deficits, and exacerbated inflammatory response. Therefore, the aim of this study was to investigate the effects of 4 weeks of RE intermittent training on the prevention and recovery from these AD-related neuropathological conditions in APP/PS1 mice.

**Methods:**

For this purpose, 6-7-month-old male APP/PS1 transgenic mice and their littermates, negative for the mutations (CTRL), were distributed into three groups: CTRL, APP/PS1, APP/PS1+RE. RE training lasted four weeks and, at the end of the program, the animals were tested in the open field test for locomotor activity and in the object recognition test for recognition memory evaluation. The brains were collected for immunohistochemical analysis of Aβ plaques and microglia, and blood was collected for plasma corticosterone by ELISA assay.

**Results:**

APP/PS1 transgenic sedentary mice showed increased hippocampal Aβ plaques and higher plasma corticosterone levels, as well as hyperlocomotion and reduced central crossings in the open field test, compared to APP/PS1 exercised and control animals. The intermittent program of RE was able to recover the behavioral, corticosterone and Aβ alterations to the CTRL levels. In addition, the RE protocol increased the number of microglial cells in the hippocampus of APP/PS1 mice. Despite these alterations, no memory impairment was observed in APP/PS1 mice in the novel object recognition test.

**Discussion:**

Altogether, the present results suggest that RE plays a role in alleviating AD symptoms, and highlight the beneficial effects of RE training as a complementary treatment for AD.

## 1. Introduction

Alzheimer’s disease (AD) is characterized by clinical symptoms, such as loss of cognitive functions, memory deficits, and progressive dysfunction of motor and behavioral activities (McKhann et al., 1984). One of the main neuropathological alterations of AD is the formation of senile plaques due the accumulation of amyloid-β (Aβ) protein which leads to synaptic transmission impairment and neuronal damage, initiating the neurodegenerative process (Ashe and Zahs, 2010; Ma et al., 2022).

The extracellular plaque formation leads to increased neuroinflammation and neuronal loss, which evolves cognitive impairment and clinical symptoms (Braak and Braak, 1991; Montine et al., 2012). In this initial phase of AD, there is still no considerable memory loss or cognitive impairment (Sasaguri et al., 2017), although signs of agitation (hyperlocomotor activity), restlessness and wandering (Cheng et al., 2014), in addition to increased levels of cortisol (Haraguchi et al., 2006), can already be observed. Interestingly, it has been reported that high levels of stress and corticosteroids may correlate with an increased risk of developing AD and may accelerate disease progression in the early-stage of AD-related dementia (Hartmann et al., 1997; Yuede et al., 2018; Lyons and Bartolomucci, 2020; Saeedi and Rashidy-Pour, 2021).

In experimental models using transgenic mice to study AD, the animals also present Aβ accumulation and plaque formation in the prefrontal cortex and hippocampus (Drummond and Wisniewski, 2017), as well as elevated plasma corticosterone levels (Green et al., 2006) and hyperlocomotion in the open field test (Jucker and Walker, 2011; Cheng et al., 2014; Wang et al., 2020). According to some studies, high levels of corticosteroids in the brain of AD animals lead to increased activation of BACE1 that, in turn, increases the activation of APP and PS1. Importantly, APP is cleaved by BACE1 (β-secretase) and subsequently by γ-secretase, forming the Aβ neurotoxic peptide (Green et al., 2006; Calvo-Rodriguez et al., 2019; Zhang H. et al., 2021).

Physical exercise, in both humans and rodents, has been associated with a lower risk of dementia and cognitive impairment in aging (Buchman et al., 2012; Hörder et al., 2018; Liu et al., 2020). Additionally, several human studies have shown the positive impact of physical exercise in individuals diagnosed with AD, in terms of memory improvement, increased attentional levels, verbal fluency, and better performance on an intelligence scale test (Palleschi et al., 1996; Arkin, 2007; Chen et al., 2016; Stephen et al., 2017; Choi et al., 2018). In animal models, physical exercise has been shown to improve spatial memory and increase neurogenesis (van Praag et al., 2005; Tapia-Rojas et al., 2016), decrease Aβ deposition and reduce Aβ plaque formation rates (Adlard et al., 2005; Kennedy et al., 2017).

Evidence indicates that physical exercise is more effective in improving AD when it is initiated before or in the early stages of Aβ deposition (Lourenco et al., 2019). When exercise is introduced into the pre-plaque phase there is less Aβ deposition. Additionally, increased neurogenesis associated with physical exercise in the early stages of the pathogenesis of AD may help improve cognitive function, thus providing a strategy for modifying or even preventing diseases such as AD (Choi et al., 2018). Furthermore, animals exposed to physical exercise have lower levels of corticosterone, along with cognitive improvements and reduction of Aβ load in the hippocampal region (Radahmadi et al., 2015).

Most studies, however, use aerobic exercises and few have investigated the effects of other modalities that may also show benefits in AD progression, such as resistance exercise (RE; Hashiguchi et al., 2020). Considering that the quality of life of the elderly population is compromised by loss of strength and endurance, muscle atrophy (sarcopenia), and greater difficulty in being physically active, RE emerges as an important strategy to improve muscle mass, muscle strength and balance, as well as functional capacity and cognitive function (Ozkaya et al., 2005; Cassilhas et al., 2007; Liu-Ambrose et al., 2012; De Frutos-Lucas et al., 2018). Moreover, RE also produces neuroprotective effects and provides benefits such as increased release of neurotrophic factors and immunomodulatory responses, stimulating neurogenesis and neuroplasticity, and improving memory (Cassilhas et al., 2007). According to Navarro et al. (2018), RE improves brain function in the elderly and can be neuroprotective, reducing the risk for the onset of AD and other dementia. When applied to 6–7-month-old APPswe/PS1dE9 transgenic mice, daily RE promotes the control of hyperlocomotion, reduction of Aβ load in the hippocampus and decreased pro-inflammatory cytokine levels (Hashiguchi et al., 2020).

Based on that, the present work aimed to evaluate the effects of RE, such as climbing a ladder with progressive overload in alternate days during 4 weeks, on the molecular (increased Aβ protein, microglia cells, plasma corticosterone levels) and behavioral (hyperlocomotion, recognition memory impairment) alterations related to AD observed in APP/PS1 mice.

## 2. Methods

### 2.1. Animals

Adult male, 6-7-month-old APPswe/PS1dE9 transgenic mice (APP/PS1) and their littermates negative for the mutations (CTRL) were provided by CEDEME (Center for the Development of Animal Models in Biology and Medicine at the Universidade Federal de São Paulo). They were housed in polypropylene home cages (41 cm × 34 cm × 16.5 cm) in a pathogen-free facility, under controlled temperature (22–23°C) and lighting (12 h light, 12 h dark; lights on at 6:45 a.m.) conditions. Appropriate food and water were available *ad libitum*. The Ethics Committee of the Universidade Federal de São Paulo approved all experiments under the protocol 9,268,250,618. Adult male APPswe/PS1dE9 transgenic mice (APP/PS1) and their littermates negative for the mutations (CTRL) with 6-7-month-old were used.

### 2.2. Experimental design

**Figure 1:**
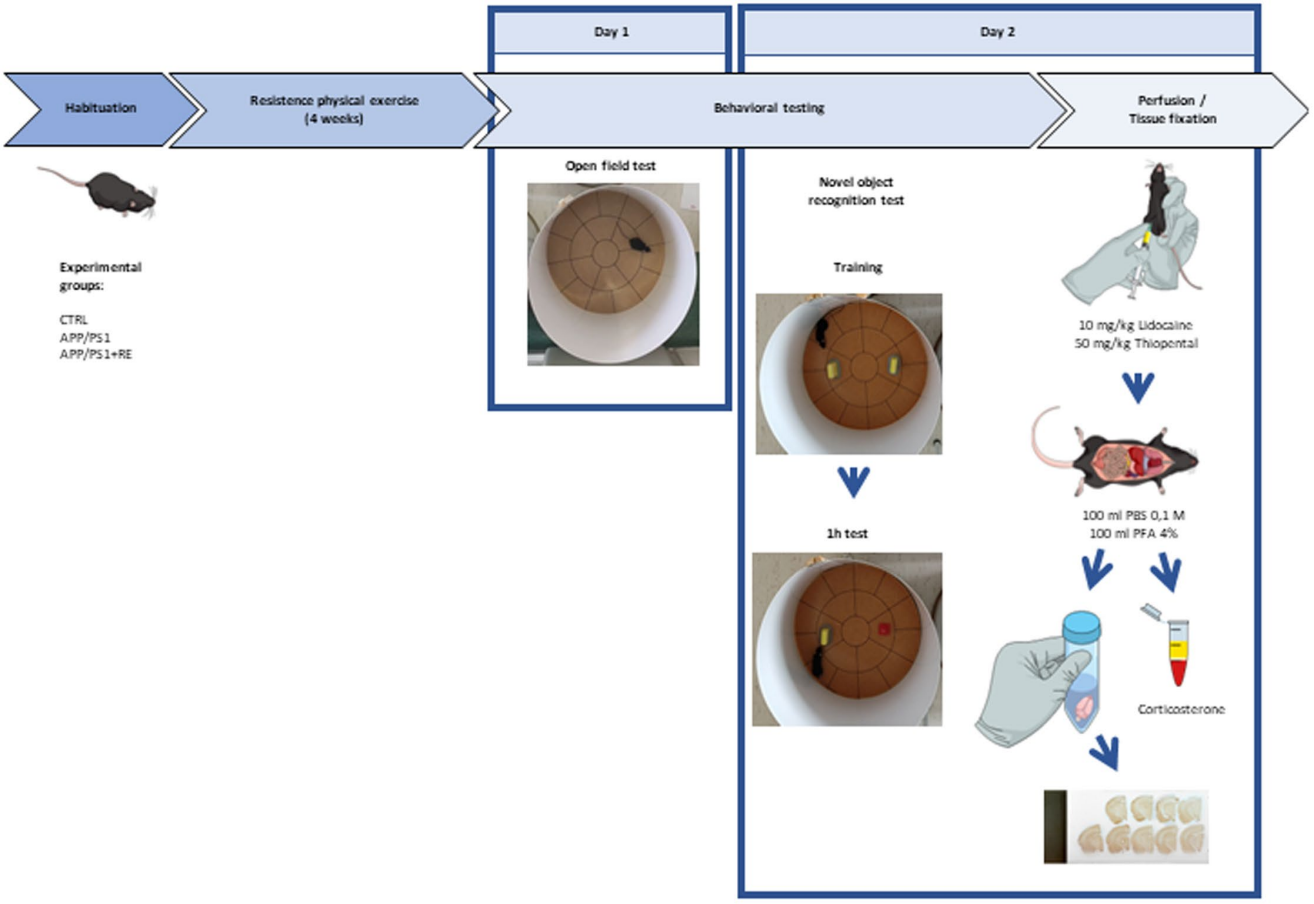
Experimental design. PBS: phosphate-buffered saline; PFA: paraformaldehyde.

Adult (6-7-month-old) male APPswe/PS1dE9 transgenic mice (APP/PS1) and their littermates negative for the mutations (CTRL) were distributed into three groups: CTRL, APP/PS1, APP/PS1 + RE. Mice from CTRL and APP/PS1 groups were kept in their home cages while animals from APP/PS1 + RE group were trained to climb a ladder with a progressive overload, every other day, for 4 weeks. One day after the last exercise training session, mice were exposed to the open field test for the evaluation of locomotor activity and anxiety-related behaviors. Twenty-four hours later, the novel object recognition test was performed. Right after the behavioral tests, animals were deeply anesthetized (10 mg/Kg lidocaine, 50 mg/Kg thiopental) and blood was sampled from the mouse atrium for the quantification of plasma corticosterone concentration by enzyme-linked immunosorbent assay (ELISA). Subsequently, mice were transcardially perfused with 100 ml of 0.1 M PBS and 100 ml 4% PFA. Brains were removed from the skull and kept in 4% paraformaldehyde (PFA) for 24 h, being transferred to a hypertonic 30% sucrose solution for cryoprotection. The brains were frozen, coronally sectioned (30 μm), and immunostained with anti-6-E10 (Aβ protein marker) and anti-Iba-1 (microglia marker) antibodies for quantitative tissue cytometry as follows below. Figure 1 provides a scheme of the experimental design.

### 2.3. Resistance physical exercise protocol

APP/PS1 mice were exposed to a RE protocol adapted from previous studies (Cassilhas et al., 2012), which consists in climbing an apparatus with a progressive overload attached to the animal’s tail. The climbing apparatus is a 110 cm high and 18 cm wide ladder, with a distance of 2 cm between each step, with an incline of 80^o^ and a shelter at the top.

The protocol includes two phases: familiarization and training. Familiarization with the device consisted of three voluntary attempts, on three consecutive days. Initially, a Coastlock Snap Swivel ring was attached to the mouse’s tail with adhesive tape (Scotch 3 M) and each animal was placed in the shelter for 60 s. In the first trial, the mouse was placed on the steps approximately 35 cm below the shelter door. In the second trial, the animal was positioned in the middle of the stairs, approximately 55 cm below the shelter door. In the third trial, the mouse climbed the entire ladder, 110 cm high. At the end, the ring was removed from the tail and the animal was placed back in its home cage.

The training phase started 2 days after familiarization. The protocol consisted of 3 sessions of progressive RE in alternate days, for 4 weeks. Therefore, the animal was exposed to 3 training sessions per week. Each training session consisted of six to eleven climbing trials, with progressive overload. In the first training session, the first two climbing trials had an overload of 75% of the animal’s body weight (weighed on the first training day). In the third and fourth trials, the overload was 90% of the animal’s body weight. In the fifth and sixth trials, the overload was 100% of the animal’s body weight. There were 60s-intervals between each of these trials. From the seventh trial on, the increase in load was 3 g at each attempt with 120 s-intervals between them. The training session was over after climbing failure or after the eleventh trial.

In the following training sessions, the trial intervals and progressive overload were the same as described above. However, the overloads were calculated in relation to the maximum load reached during the previous training session and no longer in relation to the animal’s body weight. If necessary, the animal received a light touch on the back as stimulus for climbing. Climbing failure was considered when the animal did not reach the shelter after three stimuli.

### 2.4. Open field test

One day after the last exercise training session, mice were exposed to the open field test for assessment of locomotor activity and anxiety-related behavior. The open field test was performed in a cylindric apparatus of 40 cm-diameter, 50 cm-high, and 2 cm-thick walls, as previously described (Wuo-Silva et al., 2016). The arena floor is divided into 19 sections of approximately equal dimensions: 12 peripheric sections (adjacent to the wall), 6 intermediate sections and 1 central section in three concentric circles of different radiuses (20, 14, and 8 cm, respectively).

Animals were placed in the arena for 10 min and allowed to explore it freely. All tests were video-recorded. Locomotion frequency was defined as the number of sections explored by the animal during the test; a locomotion unit was considered when the animal placed the four paws inside one of the sectors. Anxiety-like behavior was evaluated by analyzing the percentage of central crossings (ratio of the number of crossings in the intermediate or central sections to the number of total crossings multiplied by 100) performed by each animal. Decreased activity in the intermediate and central sections of the cylinder are associated to anxiogenic responses (Dao and Kovacsics, 2009).

### 2.5. Novel object recognition test

The novel object recognition test was carried out in the same open field apparatus in two sessions (training and test) as previously described (Clarke and Ludington, 2018). Training sessions were performed 24 h after the open field test. Test session was carried out 1 h after training. During the sessions animals were exposed to two objects fixed with adhesive tape in opposite sides of the apparatus. In the training session (5 min), two identical objects were presented (A and A’) and in the test session (5 min) one of these objects was replaced by a new one, with a different color and shape (B).

Object interaction was defined as smelling or touching objects with the snout or front paws. Sitting or walking around the object were not considered object interaction. The discrimination index (DI) was used to analyze the interaction time (T) between new (NT) and familiar (FT) objects. DI was calculated using the [DI = (NT − FT)/(NT + FT)]. Positive DI values indicate that the new object was more explored than the familiar one was, while negative values indicate the opposite. Zero indicates lack of preference between the objects. Animals, which had interacted less than 20 s with both objects in the training session, were excluded from the analysis since it can indicate that these animals did not show any interest for the objects, precluding the evaluation for any preference in the test session (Lueptow, 2017).

### 2.6. Plasma corticosterone concentration determination

After the behavioral tests, animals were deeply anesthetized (10 mg/Kg lidocaine, 50 mg/kg thiopental, i.p.). Blood was sampled from the mouse atrium, collected in EDTA-lined tubes and centrifuged (10 min, 1,600 *g*, 4°C). Plasma was aspirated from the pellet and stored at −20°C until analysis. Plasma corticosterone concentration was quantified using a competitive enzyme-linked immunosorbent assay (ELISA) kit (EIACORT, ThermoFisher Research). Samples were run in duplicate and compared to a standard curve according to kit instructions. Final corticosterone concentrations (nM) in the plasma of each animal were the average of duplicate reads on a standard plate reader (Invitrogen Corticosterone ELISA Kit) at 450 nm.

### 2.7. Perfusion and tissue fixation

Animals under deep anesthesia (10 mg/Kg lidocaine; 50 mg/kg thiopental, i.p.) were transcardially perfused with approximately 100 ml phosphate-buffered saline (PBS) and 100 ml of 4% PFA. The brains were removed from the skull and kept in 4% PFA for 24 h, being transferred to a hypertonic 30% sucrose solution for cryoprotection. The brains were frozen and coronally sectioned (30 μm). Sections were stored in anti-freezing solution (500 ml PBS, 500 ml H_2_O, 1.59 g NaH_2_PO_4_, 5.47 g Na_2_HPO_4_, 300 g sucrose, 300 ml ethylene glycol) at −20°C.

### 2.8. Immunohistochemistry/immunofluorescence

Free-floating hippocampal sections were washed three times for 10 minutes. Then, they were incubated with blocking buffer solution (0.1% triton [100×], and 2% of normal goat serum in PBS) for 30 min and left overnight (12 h) with the primary antibodies: Aβ plaques (mouse anti-6-E10; 1:1,000; Covance) and microglial cells (rabbit anti-Iba-1; 1:1000, Wako Chemicals). Sections were then washed and incubated with secondary antibodies (anti-mouse, 1:600, Vector, BA9200 or anti-rabbit, 1:600, Vector, BA1000) for 2 h at room temperature, followed by ABC (Avidin/Biotinylated enzyme Complex Vectastain Elite, Vector) kit incubation for 90 min and diaminobenzidine (DAB) for 5 min right after. Microglia around the plaques were identified by immunofluorescence microscopy using the anti-Iba-1 (rabbit anti-Iba-1; 1:1,000, Wako Chemicals) and anti-6-E10 (mouse anti-6-E10; 1:1,000; Covance) antibodies. On the following day, sections were incubated for 2 h with a fluorochrome-conjugated appropriate secondary antibody anti-mouse Alexa Fluor 488, anti-rabbit Alexa Fluor 488 and anti-rabbit Alexa Fluor 568 (Invitrogen) and washed in PBS. Slides were mounted and sealed with DPX.

Images were acquired with the TissueFaxs Confocal Cytometer (TissueGnostics GmbH, Vienna, Austria). Six hippocampal sections of 30 μm thickness were captured, resulting in approximately 90 fields of view/section. In each field of view, the total number of cells positive for 6-E10 or Iba-1 immunostaining was quantified with the Strata-Quest software (TissueGnostics, Vienna, Austria).

### 2.9. Statistical analysis

Statistical analysis and graphs were designed in GraphPad Prism, version 8.0. Open field test data were analyzed by Kruskal-Wallis’ test followed by Dunn’s *post hoc*. The discrimination index of the novel object recognition test, plasma corticosterone concentration, as well as the number of positive cells for 6-E10 and Iba-1 were analyzed by one-way ANOVA followed by the Dunnett’s post-test. The critical value considered to indicate significant difference between groups was 5% (*p* < 0.05).

## 3. Results

**Figure 2:**
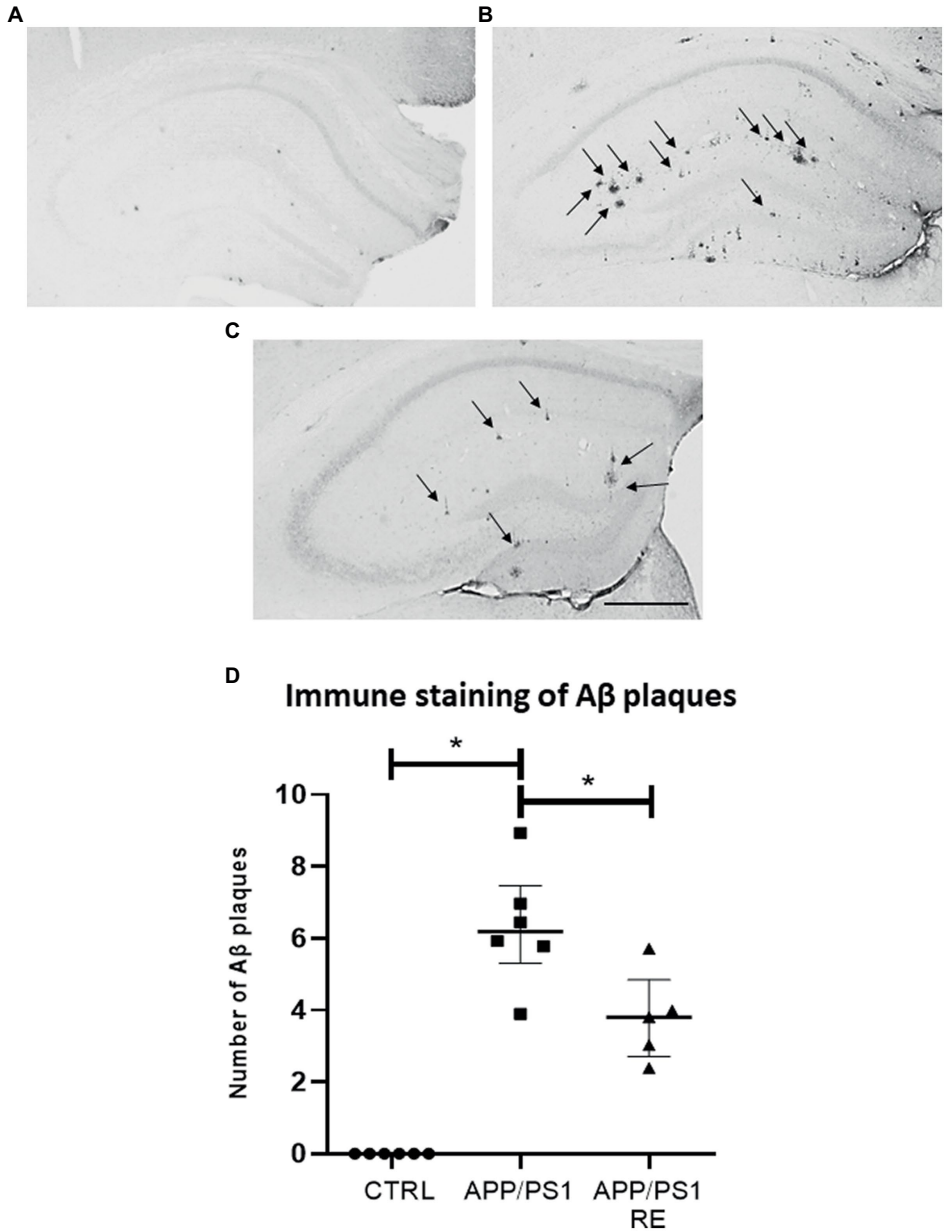
Resistance exercise reduced the number Aβ plaques in the hippocampus of APP/PS1 mice. Representative photomicrographs (scale bar = 40 μm) showing Aβ plaques (arrows) in hippocampal sections of mice from CTRL **(A)**, APP/PS1 **(B)**, and APP/PS1 + RE **(C)** groups. Graph values (mean ± standard error of the mean) represent the number of Aβ plaques labeled with 6-E10 **(D)**. Asterisks indicate significant differences between groups (*p < 0.05; one-way ANOVA followed by the Dunnett’s posttest), *n* = 5–6 animals/group.

Here, we evaluate the effect of RE in the molecular (increased Aβ protein, microglia cells, plasma corticosterone levels) and behavioral (hyperlocomotion, recognition memory impairment) alterations observed in APP/PS1 mice related to AD. As shown in Figures 2A–D, RE reduced the number of hippocampal Aβ plaques stained by anti-6-E10 antibody in APP/PS1 mice (F_2,14_ = 42.68; *p* = 0.0001; one-way ANOVA followed by the Dunnett’s post-test).

**Figure 3:**
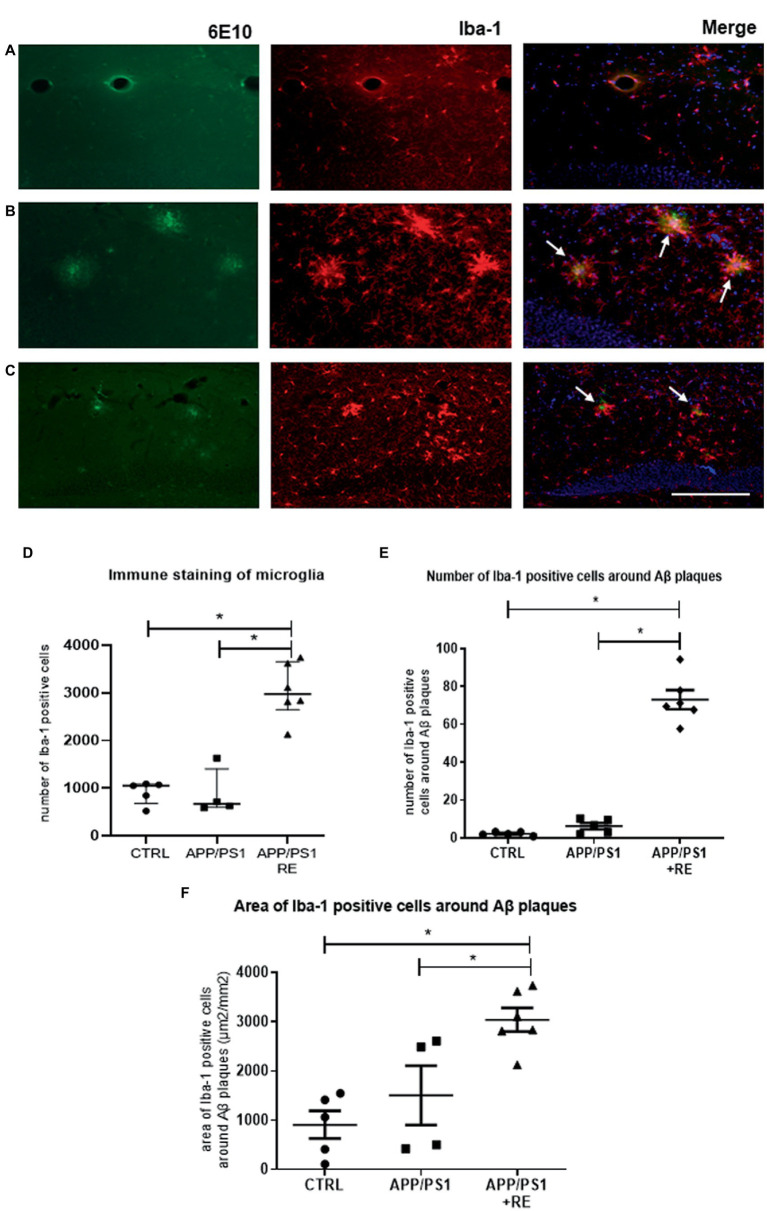
Resistance exercise increased the number and the recruitment of microglial cells around Aβ plaques in the hippocampus of APP/PS1 mice compared to CTRL or APP/PS1 sedentary animals. Representative photomicrographs (scale bar = 50 μm) of immunofluorescence of Aβ plaques (6-E10, green), microglia (Iba-1, red), and double-stained (6-E10, green + Iba-1, red) with nuclear marker (DAPI, blue) shown in hippocampal sections of mice from CTRL **(A)**, APP/PS1 **(B)**, or APP/PS1 + RE **(C)** groups, as indicated by arrows. Graph values (mean ± standard error of mean) represent the total number of Iba-1 positive cells **(D)**, the number of Iba-1 positive cells around the Aβ plaques **(E)**, and the area of Iba-1 positive cells around the Aβ plaques **(F)**. One-way ANOVA followed by the Dunnett’s post-test, *p < 0.05, *n* = 4–6 animals/group.

Furthermore, as shown by Iba-1 immune staining in Figure 3, RE increased the total number of microglial cells (D: F_2,12_ = 36.14; *p* = 0.0001; one-way ANOVA followed by the Dunnett’s post-test), as well as the number (E: F_2,13_ = 141.07; *p* = 0.0001; one-way ANOVA followed by the Dunnett’s post-test) and area (F: F_2,12_ = 10.38; *p* = 0.0024; one-way ANOVA followed by the Dunnett’s post-test) of microglial cells around Aβ plaques in the hippocampus of APP/PS1 mice, when compared to CTRL or APP/PS1 sedentary animals (one-way ANOVA followed by the Dunnett’s post-test) suggesting the regulation of neuroinflammation.

**Figure 4:**
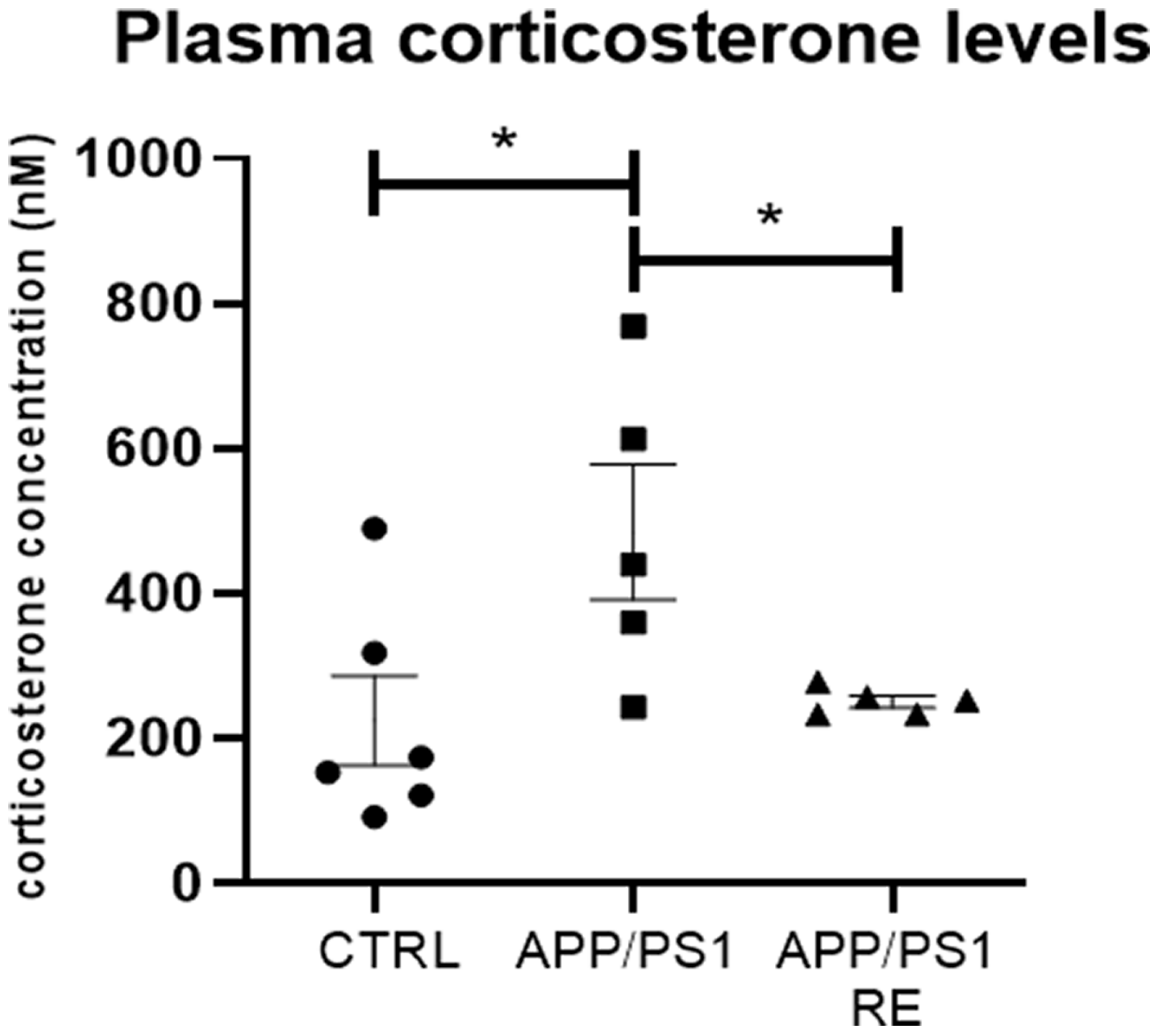
Resistance exercise decreased plasma corticosterone levels in APP/PS1 mice. Values (mean ± standard error of the mean) represent plasma corticosterone concentration (nM). One-way ANOVA followed by the Dunnett’s post-test, *p < 0.05, *n* = 5–6 animals/group.

Because augmented corticosterone activates BACE1, the enzyme responsible for the processing of APP *via* the amyloidogenic pathway and the consequent formation of Aβ peptides (Green et al., 2006; Calvo-Rodriguez et al., 2019; Zhang H. et al., 2021), it would be expected that reduced corticosterone levels as a result of RE would be responsible for the reduction of the amount of hippocampal Aβ plaques in APP/PS1 mice. Indeed, RE decreased plasma corticosterone levels in APP/PS1 (F_2,13_ = 4.792; *p* = 0.0276; one-way ANOVA followed by the Dunnett’s post-test), as shown in Figure 4.

**Figure 5:**
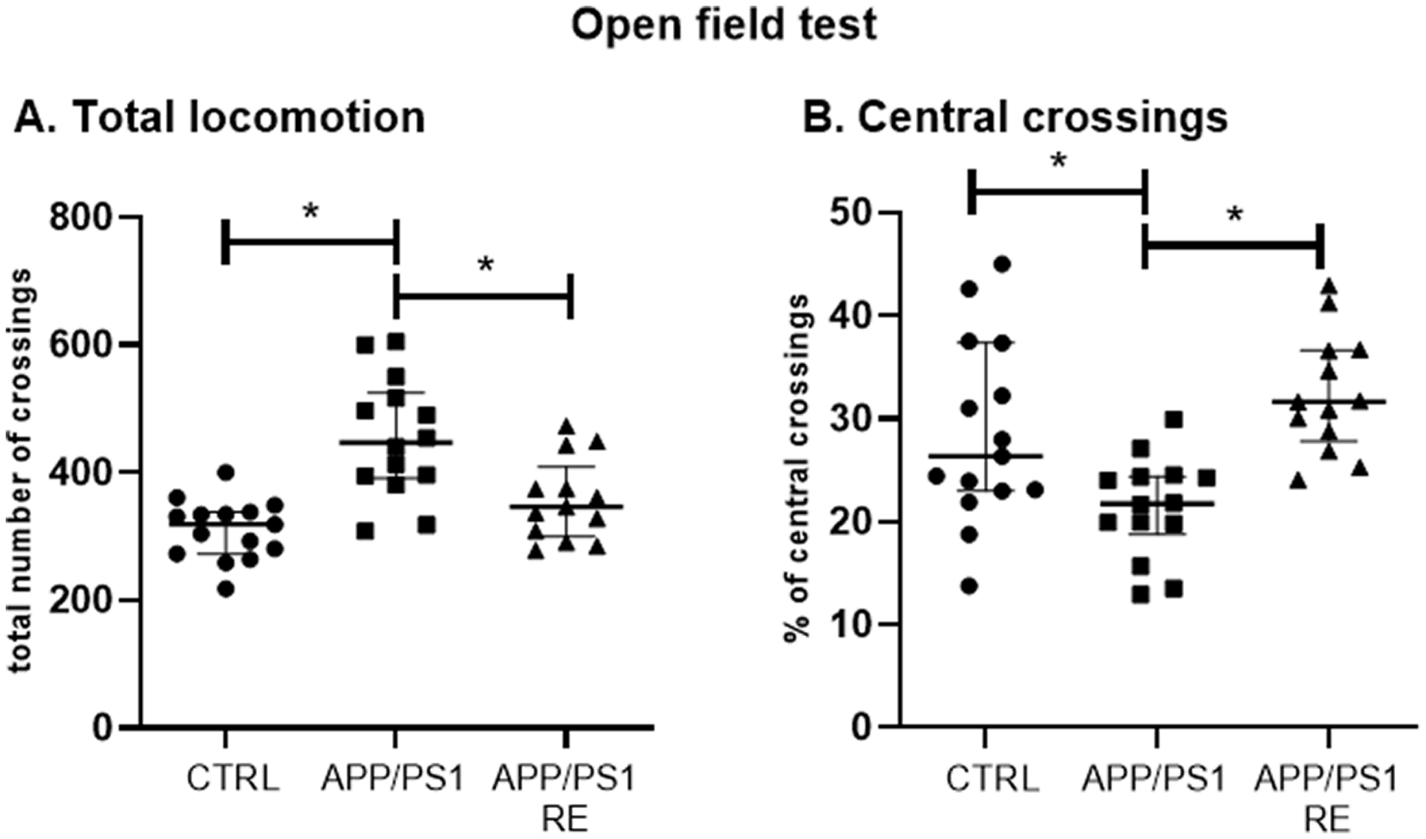
Resistance exercise prevented the occurrence of behaviors presented by the transgenic mice such as the increase in total locomotion **(A)** and the decrease in the percentage of central crossings **(B)** of APP/PS1 mice in the open field test to CTRL levels. Values (median ± interquartile range) represent the number of total crossings **(A)** or the percentage of central crossings **(B)** of mice in the open field test. Kruskal-Wallis’ test followed by the Dunn’s *post hoc*, *p < 0.05, *n* = 13–15 animals/group.

Finally, RE prevented the occurrence of behaviors presented by the transgenic mice such as the increase in total locomotion (A: H = 16.83, *p* = 0.0002) and the decrease in the percentage of central crossings (B: H = 14.95, *p* = 0.0006) of APP/PS1 mice exposed to the open field test (Kruskal-Wallis’ test followed by Dunn’s *post hoc*), as shown in Figure 5.

**Figure 6:**
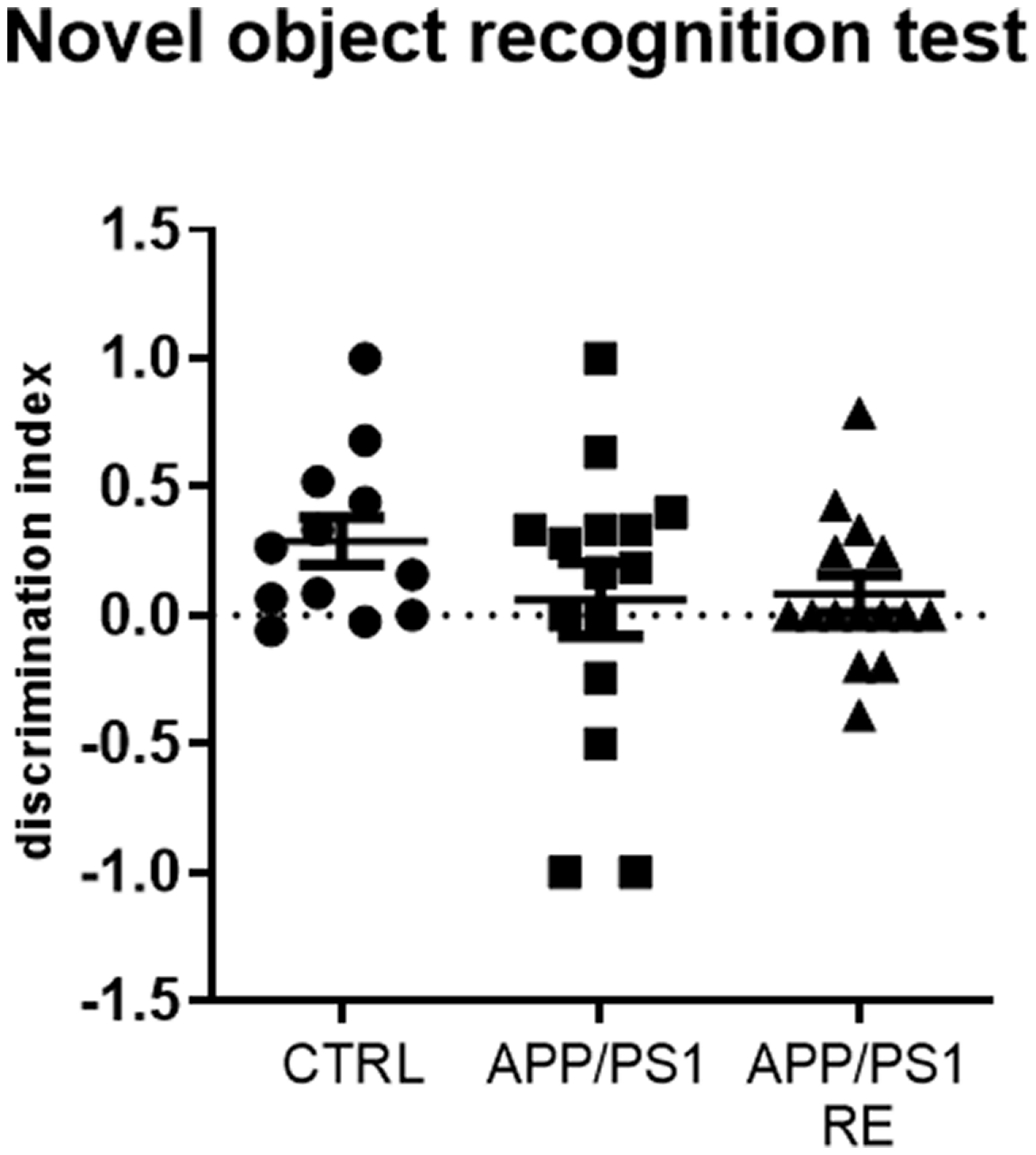
Seven-month-old APP/PS1 mice showed no impairment in short-term recognition memory. Values (mean ± standard error of the mean) represent the discrimination index for the novel object recognition test which was calculated by the ratio of the time spent on the new object minus the time spent in the familiar object to the total time spent on both objects. One-way ANOVA, *n* = 12–15 animals/group.

However, no difference between groups in the discrimination index for the novel object recognition test (F_2,39_ = 1.191; *p* = 0.3147; one-way ANOVA) was observed (Figure 6).

## 4. Discussion

Compared to their non-mutant littermates, APP/PS1 transgenic mice exhibited greater amount of hippocampal Aβ plaques (measured by the increase of 6-E10 positive cells immune stained) and higher plasma corticosterone levels, which were associated with increased locomotion and decreased central crossings in the open field test. Despite these alterations, 6–7-month-old APP/PS1 mice did not display any memory impairment in the novel object recognition test when compared with CTRL animals. Interestingly, we showed for the first time that an intermittent protocol of RE every other day for 4 weeks with progressive load, normalized these behavioral and molecular alterations observed in APP/PS1 mice to CTRL levels. In addition, RE increased the number of Iba-1 positive cells mainly around Aβ peptide deposits in the hippocampus of APP/PS1 mice, suggesting that amyloid plaques lead to the recruitment of microglial cells.

One of the first neuropathological hallmarks of AD is the presence of senile plaques formed by Aβ peptide deposition (Alzheimer, 1907; De Ture and Dickson, 2019). Recently, studies have shown a direct correlation between the reduction of Aβ load, size and quantity of Aβ plaques, and physical exercise (Brown et al., 2019; De Almeida et al., 2022). Several mechanisms by which exercise alleviates amyloid pathology have been proposed, including expression downregulation of enzymes responsible for the formation of Aβ (Alkadhi and Dao, 2018; Zhang et al., 2018), reduction of astrogliosis (Liu et al., 2020), and modulation of microglial activity (Hashiguchi et al., 2020; Liu et al., 2020). Exercise can also enhance expression of Aβ-degrading enzymes, such as neprilisin-2, insulin-degrading enzyme, and proteasome (Quan et al., 2020; Vasconcelos-Filho et al., 2021; Xu et al., 2022). Moreover, exercise may have a greater effect on amyloid pathology in the early stages of the disease (Huuha et al., 2022), suggesting that the benefits of exercise in modulating the burden of Aβ may depend on the age, and the stage of the disease (Azevedo et al., 2023). According to our results, RE reduced the number of Aβ plaques in the hippocampus of these animals, corroborating studies that also showed the clearance of Aβ after a RE program (Özbeyli et al., 2017; Liu et al., 2020; Pena et al., 2020), although others that have shown a reduction in the volume of plaques (Hashiguchi et al., 2020) or expression of Aβ peptide (Pena et al., 2020) could not find differences in the levels of the protein APP (Hashiguchi et al., 2020; Pena et al., 2020).

Another hallmark of AD is the exacerbation of a neuroinflammatory responses mediated primarily by the recruitment and stimulation of microglial cells in pro-inflammatory state (Ardura-Fabregat et al., 2017). Indeed, the presence of activated microglia in the AD brain is found in close association with amyloid plaques. In the initial processes of the disease, activation of microglia with phagocytic activity can serve to remove Aβ peptides, and protect the brain from pathogens by removing cellular debris, producing and releasing pro- and anti-inflammatory cytokines in an attempt to maintain cerebral homeostasis (Mangialasche et al., 2010; Gelman et al., 2018). However, it is known that, as the disease progresses, Aβ deposits accumulate in the hippocampus leading to increasing neuroinflammatory processes (Jardanhazi-Kurutz et al., 2010). Subsequent chronic microglial activation that occurs throughout neurodegenerative processes can become harmful, leading to failures and dysfunctions in the cytokine production as well as in the capability of phagocyting and clearing Aβ oligomers (Hernandez et al., 2010; Baik et al., 2016; Hickman et al., 2018; Zucchella et al., 2018). In an elegant study, Daria et al. showed that the clearance and phagocytic function of the amyloid plaque of the aged microglia was recovered by exposing the ancient microglia to conditioned medium of young microglia, this was sufficient to induce microglial proliferation and reduce the size of the amyloid plaque (Daria et al., 2017).

Interestingly, RE increased the number of microglial cells in the hippocampus of APP/PS1 mice. In addition, we observed that these cells are preferentially located around the plaques, and the number (Figure 3B) and area (Figure 3C) covered by Iba-1 positive cells surrounding the β-amyloid plaques were increased in APP/PS1 + RE mice, but not in the transgenic sedentary animals, corroborating another study showing that microglia around the amyloid plaques can protect the surrounding environment by forming a barrier of processes that condense the plaques (Condello et al., 2015). In fact, it has been described that exercise is capable of reducing neuroinflammation by modulating microglial activation, and consequently diminushing pro-inflammatory cytokine levels and improving the pathogenesis of AD (for review see Kelly, 2018; De Sousa et al., 2021; De Almeida et al., 2022). It has also been shown that exercise can shift activated microglia from a pro-inflammatory M1 to an anti-inflammatory M2 phenotype in a sporadic AD model (Lu et al., 2017), together with studies that showed that exercise induces microglia proliferation and increases gene expression in microglia of a pro-neurogenic phenotype (Ehninger and Kempermann, 2003; Littlefield et al., 2015). Therefore, the improvement observed in RE animals may be related to a change in exercise-triggered microglial activity, moving from a pro-inflammatory to an anti-inflammatory state. Although this issue has not been addressed here, it is plausible that RE could induce an increase in the anti-inflammatory phenotype of the microglial cells (or a decrease in the pro-inflammatory phenotype), which in turn could contribute to the decrease in Aβ load. Thus, we can speculate that it would not necessarily be associated with a decreased number of microglial cells, but rather with changes in microglial M1 and M2 phenotype patterning.

The hyperlocomotion shown by APP/PS1 mice in the open field test corroborates previous studies (Hooijmans et al., 2009; Cheng et al., 2014), and has been associated with agitation and increased locomotor activity observed in patients with AD (Chung and Cummings, 2000). These behaviors were reduced by RE, suggesting that this modality of physical exercise can improve AD-related behaviors as described in the literature (Zhou et al., 2022; Braz de Oliveira et al., 2023).

Several human studies have shown the positive impact of aerobic exercise on individuals diagnosed with AD (Choi et al., 2018; Gaitán et al., 2021). In animal models, voluntary exercise resulted in a lower deposition of Aβ in the hippocampus (Cassilhas et al., 2012; Kennedy et al., 2017), and improved spatial memory in Morris’s maze of transgenic AD mice (Tapia-Rojas et al., 2016). More particularly, also RE showed positive effects on human brain function (Borst et al., 2001; Cassilhas et al., 2007; Arazi et al., 2021; Castaño et al., 2022), and on memory deficits of animal models of AD (for review see Azevedo et al., 2023). RE is likely to exert its effects *via* mechanisms distinct from those of aerobic exercise (Barha et al., 2017; Tsai et al., 2017; Gaitán et al., 2021). For example, Cassilhas et al. 2012 showed that both aerobic and RE improves spatial memory activating distinct molecular mechanisms. Aerobic exercise modulates BDNF/TrkB and -CaMKII, whereas RE activates IGF-1/IGF-1R and AKT pathways.

Regarding the novel object recognition test, our results corroborate data from the literature showing that 6-7-month-old APP/PS1 animals present no impairment in recognition memory (Cheng et al., 2014). Studies indicate that deficits in recognition memory increase with age in APP/PS1 mice (Jardanhazi-Kurutz et al., 2010; Qiu et al., 2016; Georgevsky et al., 2019). In addition to age, the type of protocol used may also influence the behavioral response. One of the factors that must be controlled is the level of stress that animals were exposed to, which can be assessed by measuring glucocorticoid levels (Mormède et al., 2007). As shown before, both stress and AD increase the levels of circulating glucocorticoids (Umegaki et al., 2000). Furthermore, epidemiologic clinical studies suggest the use of the analysis of long-term cortisol measurements as a biomarker to help the diagnosis of people with AD (Ennis et al., 2017). A previous study showed that 9-month-old APP/PS1 transgenic mice exhibit high plasma corticosterone levels (Zhang S. Q. et al., 2021). Moreover, chronic administration of corticosterone accelerates cognitive impairment and increases Aβ plaque formation in the hippocampus and prefrontal cortex of these animals (Zhang S. Q. et al., 2021). Other animal models for the study of AD, such as 3 × TgDA, also show increased plasma levels of corticosterone when compared with the control group (Green et al., 2006). In order to investigate whether the RE protocol used was stressful for the animals, we measured the plasma corticosterone levels. According to our hypothesis, RE was not stressful for the animals but decreased the plasma corticosterone levels presented by APP/PS1 mice.

Since increased levels of corticosterone induces activation of BACE1, the enzyme responsible for processing APP *via* the amyloidogenic pathway and consequent formation of Aβ peptides (Green et al., 2006; Calvo-Rodriguez et al., 2019; Zhang H. et al., 2021), we speculate that the decrease in corticosterone levels induced by RE contributes to the decrease in hippocampal Aβ plaques of APP/PS1 mice. Together, these factors may have contributed to the normalization of locomotor behavior in APP/PS1 animals subjected to RE.

Overall, this study highlights the beneficial effects of RE training as a complementary treatment of AD, a topic that has recently been addressed and reviewed by our research group (Azevedo et al., 2023).

## Data availability statement

The original contributions presented in the study are included in the article/supplementary material, further inquiries can be directed to the corresponding authors.

## Author contributions

HC, DR, HU, and BM designed the study and wrote the manuscript. HC, DH, and DR carried out the experiments and data analysis. HC, DR, MM, HU, and BM contributed to the article’s literature search. HC, DR, DH, and CG contributed to the conception. HC, DR, DH, TG, MM, CG, DS, RA, HU, and BM contributed to the revision of the manuscript. All authors contributed to the article and approved the submitted version.

## Funding

This work was supported by Fundação de Amparo à Pesquisa do Estado de São Paulo (FAPESP grants 2022/00249-8, 2018/07366-4, 2021/01478-8, and 2018/17504-5), Coordenação de Aperfeiçoamento de Pessoal de Nível Superior, Brazil (CAPES; Finance Code 001), (CAPES-PRINT # 88881.310490/2018-01), and Conselho Nacional de Desenvolvimento Científico e Tecnológico (CNPq processes #302608/2019-2, #408676/2018-3, #312904/2021-5, 406396/2021-3, #302689/2022-2, and 308012/2021-6). HU also acknowledges grant support by the National Institute of Science and Technology in Regenerative Medicine (*INCT*-*REGENERA*), Brazil.

## Conflict of interest

HU is a scientific advisor of TissueGnostics (Vienna, Austria).

The remaining authors declare that the research was conducted without any commercial or financial relationships that could be construed as a potential conflict of interest.

## Ethics statement

The animal study was reviewed and approved by CEUA UNIFESP 9268250618.

## Publisher’s note

All claims expressed in this article are solely those of the authors and do not necessarily represent those of their affiliated organizations, or those of the publisher, the editors and the reviewers. Any product that may be evaluated in this article, or claim that may be made by its manufacturer, is not guaranteed or endorsed by the publisher.
